# MGMT unmethylation and high levels of CD47 and TIGIT indicate a poor prognosis in adult diffuse gliomas

**DOI:** 10.3389/fimmu.2024.1323307

**Published:** 2024-02-09

**Authors:** Lingbo Ma, Yi Shi, Chang Li, Bin Deng, Jinfang Jiang, Yuwen Cao, Lianghai Wang, Hongyan Li

**Affiliations:** ^1^ NHC Key Laboratory of Prevention and Treatment of Central Asia High Incidence Diseases, The First Affiliated Hospital/Shihezi University School of Medicine, Shihezi, China; ^2^ Department of Pathology, First Affiliated Hospital of Xinjiang Medical University, Urumqi, China; ^3^ Department of Cardiothoracic Surgery, Zhuhai People's Hospital, Zhuhai Hospital Affiliated with Jinan University, Zhuhai, China; ^4^ Department of Medical Record Statistics, Northeast Yunnan Regional Central Hospital, Zhaotong, China; ^5^ Department of Neurology, People’s Hospital of Xinjiang Uygur Autonomous Region, Urumqi, China

**Keywords:** adult-type diffuse glioma, IDH mutant, p*TERT* mutation, MGMT methylation, CD47, TIGIT

## Abstract

**Introduction:**

In 2021, the World Health Organization published a new classification system for central nervous system tumors. This study reclassified the adult diffuse glioma (ADG) into astrocytoma, oligodendroglioma, and glioblastoma (GBM) according to the new tumor classification.

**Methods:**

The association of TERT promoter (pTERT) mutation, MGMT methylation, and CD47/TIGIT expression with patient prognosis was investigated.

**Results:**

Immunohistochemical analysis showed that the expression levels of CD47 and TIGIT in tumor tissues were significantly higher than those in normal brain tissues. CD47 levels were higher in GBM and grade 4 astrocytoma tissues. TIGIT expression was also higher in patients with GBM. The high expressions of CD47, TIGIT, and CD47/TIGIT were positively correlated with *MGMT* unmethylation but not p*TERT* mutation. Moreover, *MGMT* unmethylation was associated with poor overall survival in astrocytoma. High CD47, TIGIT, and CD47/TIGIT levels were associated with significantly reduced survival in ADG and GBM. GBM, *MGMT* unmethylation, and high CD47 expression were independent prognostic factors for overall survival in ADG.

**Discussion:**

Collectively, these results showed that the *MGMT* unmethylation and high levels of CD47 and TIGIT are associated with a poor prognosis in ADG. Patients with high CD47 and TIGIT expression may benefit from anti-CD47 and TIGIT immunotherapy.

## 1 Introduction

According to the Global Cancer Statistics, brain and nervous system tumors accounted for 310,000 new cases worldwide and 250,000 deaths (1). The survey report of the National Cancer Center of China showed that nervous system tumors caused 70,000 deaths in 2020, of which glioma accounted for about 60% (2). In recent years, considerable progress has been made in treating gliomas by surgical resection, radiotherapy, and chemotherapy. However, the clinical efficacy of these conventional treatments is still far from satisfactory. Studies have revealed various driver genes in glioma that may be closely associated with prognosis and have demonstrated that prognosis cannot be predicted exclusively based on morphological characteristics. In 2021, the World Health Organization (WHO) published the Classification of Tumors of the Central Nervous System (CNS), fifth edition, which introduced a new tumor classification system (3). The system involves an integrated diagnostic model based on morphological characteristics and gene expression levels in tumors, serving as a guide for clinical diagnosis, treatment, and prognosis.

Epigenetic silencing of the DNA repair enzyme O^6^-methylguanine-DNA methyltransferase (MGMT) has emerged as a prognostic and predictive marker in patients with glioblastomas (GBM) (4). However, the diagnostic criteria for GBM have shifted from purely histomorphological to molecular characteristics. In the 2021 WHO classification of tumors of the central nervous system, GBM in adult-type diffuse gliomas (ADG) is defined as the absence of mutations in the isocitrate dehydrogenase gene (i.e., *IDH* wild-type) (3). Meanwhile, patients with *IDH* wild-type and *TERT* promoter (p*TERT*) mutant gliomas in the new classification are also diagnosed with GBM. Studies on the status of p*TERT* and *MGMT* in GBM have also been reported (5), but there are few studies on p*TERT* mutations and *MGMT* methylation status in other types of ADG in the new classification.

Immune checkpoint inhibitors (ICIs) are a promising treatment for glioma (6, 7). Programmed death-1 (PD-1)/programmed death ligand-1 (PD-L1) inhibitors are the most widely used inhibitors in immunotherapy. Although ICIs offer hope to many patients with advanced malignancies, their efficacies range between 15% and 60%, with many patients showing no benefit (8, 9). Gliomas, especially GBMs, are immunosuppressive tumors. Therefore, the identification of relevant biomarkers, as well as effective immunosuppressants for treating gliomas, are important areas of research. Differentiation cluster 47 (CD47), a glycoprotein widely expressed on the cell surface, regulates tumor invasion and metastasis (10). CD47 inhibitors have been tested in clinical trials for the treatment of ovarian cancer, colorectal cancer, and leukemia (11, 12). However, studies related to their effect on glioma remain limited. T cell immune receptor with Ig and ITIM domains (TIGIT) is a novel checkpoint inhibitor molecule expressed on a variety of immune cells, including T cells, regulatory T cells (Tregs), and natural killer cells (NK) (13). They are highly expressed in colon cancer, multiple myeloma, breast cancer, and prostate cancer and are closely related to prognosis (14). Targeting TIGIT may restore T-cell function, leading to antitumor effects (15). Therefore, in ADG, exploring the expression levels of CD47 and TIGIT and their prognostic relationships, as well as the relationship between p*TERT* mutations and *MGMT* methylation status and prognosis, may provide some valuable clues for the therapy of ADG.

## 2 Materials and methods

### 2.1 Human tumor samples

The study was approved by the Ethics Committee of the First Affiliated Hospital of Shihezi University, and signed informed consent forms were obtained from patients. Glioma and normal brain tissues were acquired from participants who had received excision surgery from May 2012 to October 2021. Patients who had undergone previous adjuvant radiotherapy or chemotherapy were excluded. A total of 125 patients diagnosed with astrocytic and oligodendroglial tumors according to the Classification of Tumors (fourth edition and fourth edition revision) were included in the study. These 125 cases included 35 cases of diffuse astrocytoma, 35 cases of anaplastic astrocytoma, 42 cases of GBM, 8 cases of oligodendroglioma, and 5 cases of anaplastic oligodendroglioma. Reclassification of ADG was performed by two neuropathologists who independently reviewed histological sections and various test findings of this group of patients according to the diagnostic criteria of the WHO Classification of Tumors (fifth edition). Patients with gliomas were followed up starting from October 1, 2016. Patient survival data were obtained via outpatient examinations and telephone follow-ups. Overall survival (OS) was defined as the duration from surgery to death or the last follow-up. The review deadline was January 15, 2023.

### 2.2 Immunohistochemical analysis

Tissue samples were fixed in 10% neutral formalin and embedded in paraffin. All tissue samples were stained with antibodies against IDH1 R132H (H9), CD47 (mouse anti-human, ready-to-use type, OT13B10; Beijing Zhongshan Co.), and TIGIT (rabbit anti-human, 1:100, BLR047F; Abcam). A Leica Bond HRP Poly Kit (Shanghai Gene Company) was used following standard procedures. All immunohistochemical (IHC) sections were scanned using a digital pathology slide scanner (KF-PRO-005; Ningbo Jiangfeng Biological Information Technology Co., Ltd.). The scanned images were scored by two neuropathologists. Ten different tumor areas were selected from each specimen, and the percentage and intensity of positive tumor cells were observed and recorded (16). Positive percentage scores were 0 (0-5%), 1 (6-25%), 2 (26-50%), and 3 (more than 51%); positive intensity according to 0 (no staining), 1 (light brown), 2 (brown), 3 (dark brown); the two scores were multiplied and considered the final score. The scoring results of two pathologists were summarized, and the samples with inconsistent scoring results were discussed and scored again. All cases had a final score greater than the median and were considered high expression; ≤ the median is low expression. Among the 115 ADG cases, the median CD47 and TIGIT score was 2, and a score greater than 2 was a high expression. A score of less than or equal to 2 is a low expression.

### 2.3 Detection of IDH1/2 mutation

A DNA Extraction Kit (QIAamp Paraffin Tissue) was used to extract genomic DNA from all glioma tissues. *IDH1* R132H mutations were detected by qPCR (7500fast, ABI) using the detection reagents from Beijing Fanshengzi Gene Technology Co, Ltd. The experiment was performed according to the manufacturer’s instructions, and ΔCT ≤ 8 was interpreted as positive. First-generation sequencing was performed in cases showing inconsistent results between IHC and qPCR, in cases showing negative results by both methods and in patients younger than 55 (17). For gene sequencing, primers for *IDH1* (R132H, R132C, and R132S) and *IDH2*R172K were designed by Shanghai Bioengineering Co., Ltd (18). Primer sequences are shown in the **Supplementary Material**. Amplification and sequencing were completed by the Sequencing Department of Shanghai Bioengineering Co., Ltd.

### 2.4 Detection of pTERT mutant and MGMT methylation

The qPCR reagents for detecting the p*TERT* mutation (C228T and C250T) were provided by Beijing Fanshengzi Gene Technology Co., Ltd. and were used according to the instructions provided. Values of ΔCT ≤ 9 were interpreted as positive; otherwise, the experimental results were invalid. *MGMT* methylation reagents were obtained from Shanghai Gene Corporation. First, bisulfite conversion was performed, and then qPCR amplification to detect fluorescence signals. When ΔCT ≤ 7, the methylation level of the *MGMT* gene in the sample was ≥1%, and *MGMT* methylation was positive.

### 2.5 Fluorescence *in situ* hybridization

To evaluate the gene copy number status, fluorescence *in situ* hybridization (FISH) was used to analyze chromosome arms 1p and 19q, *CDKN2A* (Vysis), *EGFR*, chromosome 7, and chromosome 10 (Anbiping). The pretreatment kit was obtained from Beijing Yakangbo Biotechnology Co., Ltd. The experiment was performed according to the instructions accompanying each reagent. In brief, green and orange fluorescence signals were observed under a fluorescence microscope (ZEISS Image A2) with appropriate filters. The detection thresholds for 1p/19q co-deletion (19), CDKN2A deletion (20), EGFR amplification, chromosome 7 polysomy, and chromosome 10 monosomy (21) are detailed in **Supplementary Material**.

### 2.6 Statistical analysis

Statistical analyses were performed using IBM SPSS Statistics, version 26.0. Normally distributed data are expressed as the mean ± standard deviation, while count data are expressed as a frequency or percentage. Group comparisons were performed using the chi-square test or Fisher’s test. Survival rates were evaluated using the Kaplan-Meier method, and differences between the survival curves for each group were assessed using the log-rank test. Hazard ratios (HRs) and 95% confidence intervals (CIs) were estimated using Cox proportional hazards regression models. Statistical significance was set at *P* < 0.05.

## 3 Results

### 3.1 IHC combined with qPCR and sequencing could accurately detect *IDH1/2* mutation

**Figure 1:**
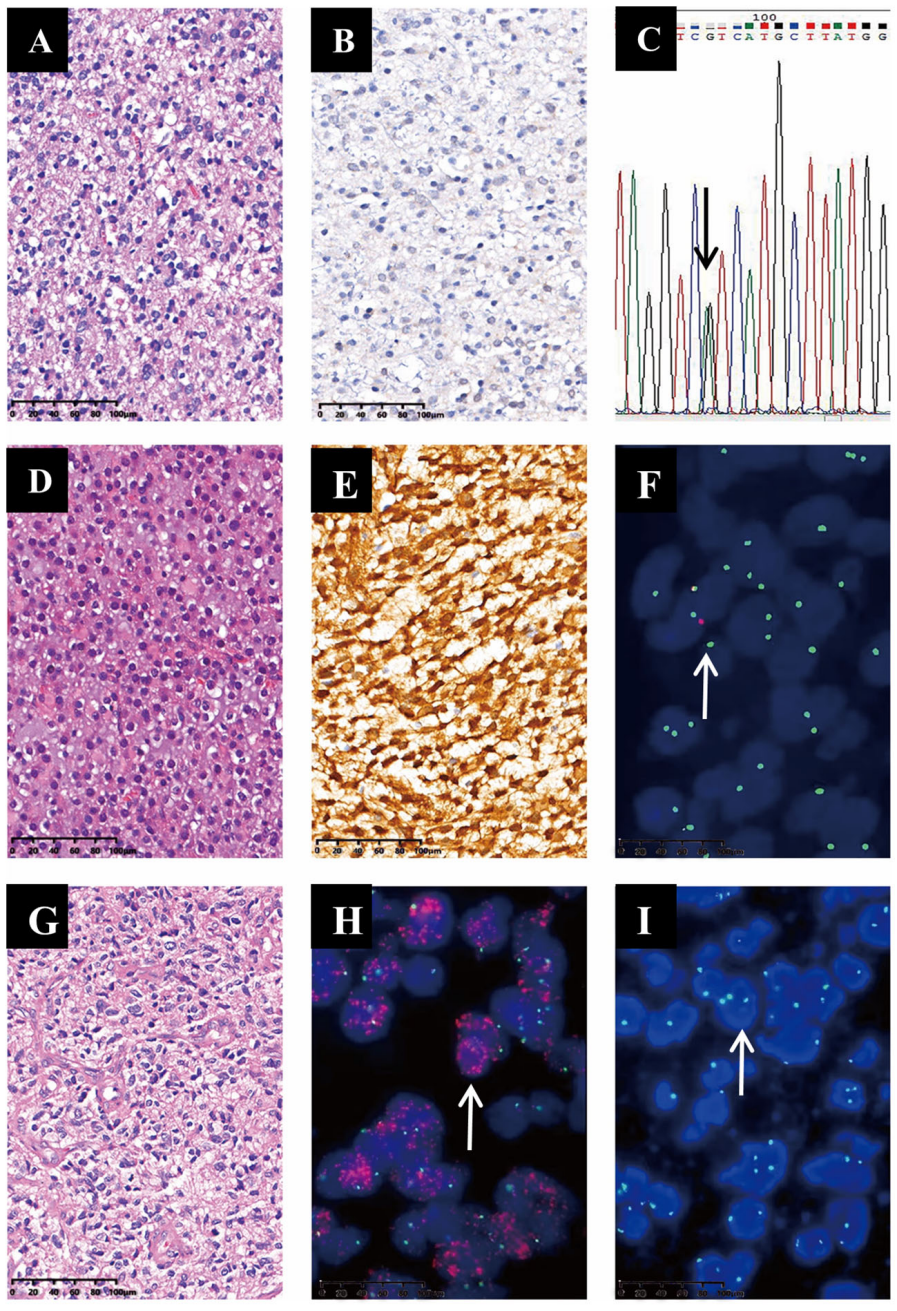
Three representative cases of ADG were evaluated by HE, IHC, and molecular tests. Case 1 was a 49-year-old male; HE **(A)**; IDH1 R132H negative (200×magnification) **(B)**; sequencing results indicated *IDH1* R132H, 395 G>A **(C)**. Case 2 was a 36-year-old male; HE morphology was characterized as a low-grade pattern **(D)**; IDH1 R132H positive (200×magnification) **(E)**; FISH showed *CDKN2A* homozygous deletion (loss of red *CDKN2A* signals, white arrow) (1000×magnification) **(F)**. Case 3 was a 46-year-old female; HE showed obvious cellular atypia and proliferation of blood vessels (200×magnification) **(G)**; FISH test showed *EGFR* amplification (red *EGFR* signals are seen, white arrow) **(H)**, chromosome 7 gain (green chromosome 7 signals are seen, white arrow; 1000×magnification) **(I)**.

IHC results indicated that the positive rate for IDH1 R123H in 125 glioma cases was 49.6% (62/125), while qPCR and first-generation sequencing showed that the mutation rate of the *IDH1/2* gene was 46.4% (58/125). Among 42 cases successfully sequenced for IDH1, seven were positive for R132H, while R132C and R132S were not detected. In contrast, *IDH2* R172K mutation was not seen among the 39 successfully sequenced cases. The consistency rate of IDH1/2 detection by IHC, qPCR, and sequencing techniques was 85.6% (107/125), and discordance between IHC and qPCR results was evident in 18 cases. Eleven cases were IHC-positive but *IDH1* wildtype by qPCR and sequencing. Seven samples were negative for IDH1 R132H via IHC, while *IDH1* mutations were detected via the other two methods (**Figures 1A–C**). The concordance rate between qPCR and sequencing techniques was 100%.

### 3.2 Reclassification of ADG

In 125 gliomas, we detected the *IDH1/2* mutation in 58 cases, co-deletion of chromosome arms 1p and 19q (1p/19q co-deletion) in 22 cases (22/125, 17.6%), *IDH1/2* mutation and 1p/19q codeletion in 16 cases, wildtype *IDH1/2* with 1p/19q- co-deletion in six patients, and the p*TERT* mutation in 64 cases (64/125; 51.2%), with C228T and C250T mutation rates of 76.56 (49/64) and 23.44% (15/64), respectively. Histomorphology and molecular detection results revealed eight cases each of grades 2 and 3 oligodendroglioma carrying mutant *IDH* with the 1p/19q-codeletion; 18, 11, and 13 cases of grades 2, 3, and 4 astrocytoma carrying mutant *IDH*. One case of grade 2 astrocytoma with homozygous deletion of *CDKN2A*, according to the new tumor classification, this patient should be IDH-mutant, grade 4 (**Figures 1D–F**). In 45 of 67 wild-type *IDH* samples, high cell density and frequent mitotic signs with angiogenesis or necrosis were observed, with one case of *EGFR* amplification and chromosome 7 polysomy (**Figures 1G–I**). Among 22 cases of low-grade glioma (LGG) with wild-type *IDH*, 12 patients had a median age of 53 (37–73) and were diagnosed with molecularly characterized GBM (10 cases had a p*TERT* mutation; two carried the *EGFR* amplification, one of which showed a chromosome 7 gain) (22). The other 10 LGGs with wild-type *IDH* and p*TERT* had no *EGFR* amplification or chromosome 7 or 10 changes. After combining the molecular markers with clinical characteristics and follow-up information of the patients, 7 cases were low-grade pediatric glioma, and the other three cases were diffuse midline glioma with the H3K27 variant. Therefore, they were excluded from this study.

### 3.3 Clinical and molecular information of ADG

**Figure 2:**
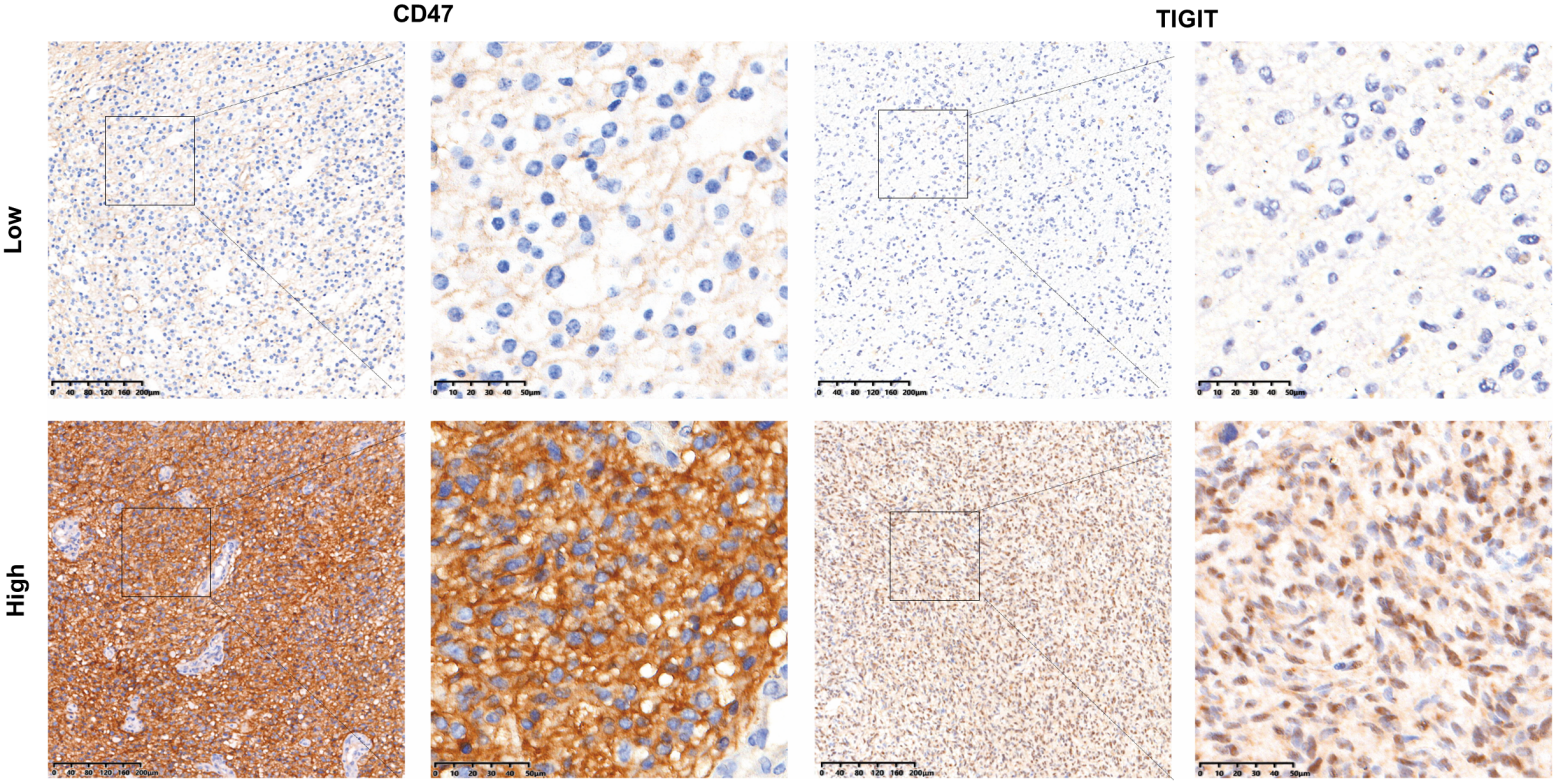
CD47 and TIGIT expression in ADG tissues. (100× and 400×magnification).

The results of HE, IHC, and molecular tests for 115 cases with ADG were reviewed based on the new tumor classification by neuropathologists. The cases were reclassified as follows: 42 (23 males and 19 females; average age 45.26 ± 12.01) cases of astrocytoma carrying mutant *IDH* where 17, 11, and 14 cases were grades 2, 3, and 4, respectively; 16 (12 males and four females; average age 46.19 ± 12.12) cases of oligodendroglioma carrying mutant *IDH* with 1p/19q-codeleted, where 8 cases each were grades 2 and 3; and 57 (31 males and 26 females; average age 54.75 ± 13.08) cases of GBM, *IDH*-wildtype. Clinical and molecular information of the 115 ADG are shown in **Supplementary Table 1**. p*TERT* mutations and *MGMT* methylation accounted for 55.7% (64/115) and 61.7% (71/115) of ADG tissues, respectively. IHC results for CD47 and TIGIT revealed high and low expression (**Figure 2**) for CD47 in ADG in 47.0% (54/115) and 53.0% (61/115) of cases, respectively; high and low TIGIT expression in ADG was observed in 42.6% (49/115) and 57.4% (66/115) of cases, respectively.

### 3.4 p*TERT* mutation and *MGMT* methylation status in ADG

The frequency of mutant p*TERT* in GBM was significantly higher than that in astrocytoma (*P* < 0.0001) (**Supplementary Table 2**). p*TERT* mutations and *MGMT* methylation were detected in grade 2 and 3 oligodendroglioma. The rate of *MGMT* methylation in astrocytoma was significantly higher than that in GBM (*P* < 0.0001). In addition, levels of *MGMT* methylation in grade 2 astrocytoma tissue were higher than in grades 3 and 4 (*P* = 0.014).

### 3.5 Overexpression of CD47 and TIGIT in ADG tissues

**Figure 3:**
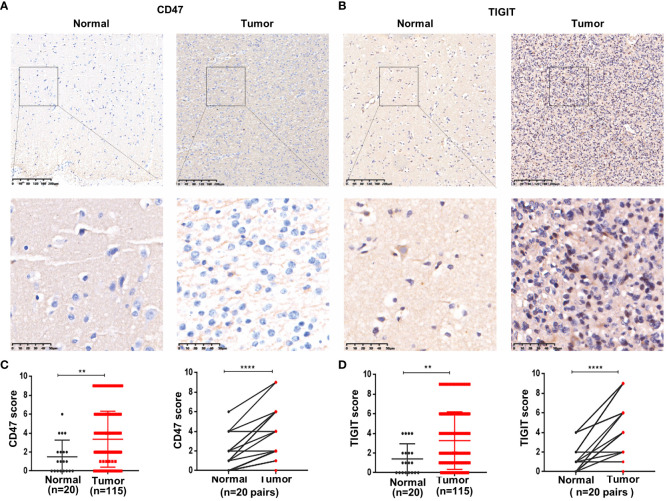
Overexpression of CD47 and TIGIT in ADG tissues. Representative images of CD47 and TIGIT in ADG and matched adjacent tissues (100× and 400×magnification) **(A, B)**. Statistical of CD47 **(C)** and TIGIT **(D)** expression in ADG and normal brain tissues. **P<0.01, ****P<0.0001.

IHC was used to detect CD47 and TIGIT expression in ADG tissues. The expression levels of CD47 and TIGIT in tumor tissues (n = 115) were significantly higher than those in normal brain tissues (*P* = 0.0073, *P* = 0.0064) (**Figure 3**). Twenty ADG tumors with matched normal brain tissues were further analyzed; the expression levels of CD47 and TIGIT in tumor tissues were higher than those in matched adjacent normal brain tissues (*P* < 0.0001). The expression of CD47 in GBM was higher than that in astrocytoma and oligodendroglioma (*P* = 0.004) (**Supplementary Table 3**) and was higher in astrocytoma grade 4 than that in grade 2 or 3 (*P* = 0.006). There were no significant differences in the expression levels of TIGIT among the three subtypes or between astrocytoma and oligodendroglioma grades 2, 3, and 4 and grades 2 and 3.

### 3.6 Relationships between CD47 and TIGIT expression and the clinicopathological characteristics of patients with ADG

CD47 expression was not correlated with sex or p*TERT* mutations (**Table 1**); however, CD47 expression was higher in patients older than 50 years of age (*P* < 0.05) and in patients classified as GBM (*P* < 0.05) and was significantly associated with grade 4 in astrocytoma, whereas high expression of CD47 and TIGIT were related to the *MGMT* unmethylation status, respectively (*P* < 0.05). High CD47 expression was positively correlated with high TIGIT expression (*P* < 0.05). High TIGIT expression was not associated with age, sex, astrocytoma grade, or the p*TERT* mutation status. However, TIGIT expression was higher in patients with GBM (*P* < 0.05). Dual CD47/TIGIT high expression was not correlated with age, sex, astrocytoma of all grades, or *pTERT* mutations. However, dual CD47/TIGIT high expression was higher in patients with GBM and associated with *MGMT* unmethylation (*P* < 0.05).

**Table 1:** Correlation between clinicopathological characteristics and TIGIT/CD47 expression in ADG cases (n=115).

Variable	CD47			TIGIT			CD47/TIGIT		
	High	Low	*P* value	High	Low	*P* value	High	Others	*P* value
Age									
≤50	22	37	0.033	27	32	0.483	16	43	0.968
>50	32	24		22	34		15	41	
Sex									
M	26	31	0.775	24	33	0.914	16	41	0.790
F	28	30		25	33		15	43	
Subtypes									
AS/OL	19	39	0.002	19	39	0.031	9	49	0.005
GBM	35	22		30	27		22	35	
AS									
2/3	6	22	0.002	10	18	1.000	4	24	0.266
4	10	4		5	9		4	10	
p*TERT*									
Mut	25	39	0.057	26	38	0.630	14	50	0.169
WT	29	22		23	28		17	34	
*MGMT*									
Met	26	45	0.005	23	48	0.005	12	59	0.002
Unmet	28	16		26	18		19	25	
TIGIT									
High	31	18	0.003						
Low	23	43							

M, male; F, female; ADG, adult-type diffuse gliomas; AS, astrocytoma, IDH-mutant; OL, oligodendroglioma, IDH-mutant and 1p/19q-codeleted; GBM, glioblastoma, IDH-wildtype; Mut, mutant; WT, wildtype; Met, methylation.

### 3.7 *MGMT* unmethylation and high levels of CD47 and TIGIT are associated with poor prognosis in ADG and subtypes

**Figure 4:**
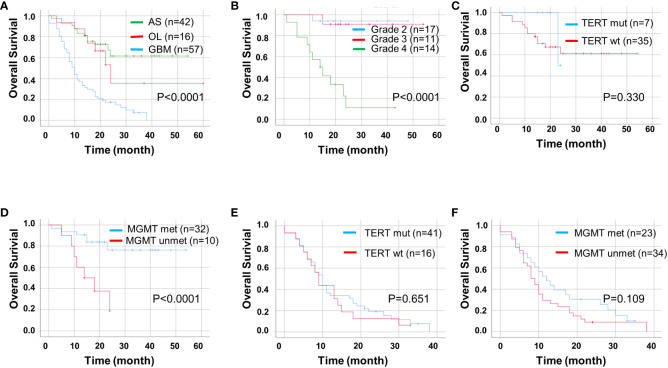
Kaplan-Meier curves of OS for ADG (n = 115). OS for ADG **(A)** and astrocytoma, IDH⁃ mutant **(B)**, and for p*TERT* mutant and *MGMT* methylation status in astrocytoma **(C, D)** and GBM **(E, F)**. AS, astrocytoma; OL, oligodendroglioma; GBM, glioblastoma; pTERT, TERT promoter; mut, mutant; wt, wildtype.

Next, we used univariate and multivariate Cox regression analyses to identify prognostic factors in patients with ADG in **Table 2**. Univariate analyses showed that age, tumor type, *MGMT* unmethylation, CD47, and high TIGIT expression were all associated with OS, whereas gender, tumor location, and p*TERT* mutations were unrelated to OS. Multivariate analysis indicated that GBM, *MGMT* unmethylation, and high CD47 expression were independent prognostic factors for OS. Furthermore, we found that groups with GBM and astrocytoma grade 4 had a lower OS than the other groups (*P* < 0.0001, **Figures 4A, B**). For pTERT mutation and MGMT methylation in astrocytoma and GBM, only the *MGMT* unmethylated group showed a shorter OS in astrocytomas (*P* < 0.0001, **Figures 4C–F**).

**Table 2:** Univariate and multivariate analyses in ADG cases (n=115).

Variable	N %	Univariate analysis		Multivariate analysis	
		HR (95% CI)	*P* value	HR (95% CI)	*P* value
Age					
≤50	59 (51.3)	2.033 (1.264-3.269)	0.003	1.336 (0.801-2.229)	0.267
>50	56 (48.7)				
Sex					
M	57 (49.6)	1.256 (0.789-1.998)	0.336		
F	58 (50.4)				
Location					
PR/TM	72 (62.6)	1.399 (0.876-2.233)	0.160		
Others	43 (37.4)				
Subtypes					
AS/OL	58 (50.4)	4.503 (2.672-7.590)	0.000	3.014 (1.644-5.527)	0.000
GBM	57 (49.6)				
p*TERT*					
Mut	64 (55.7)	1.575 (0.974-2.546)	0.064		
WT	51 (44.3)				
*MGMT*					
Met	71 (61.7)	3.267 (2.025-5.270)	0.000	1.740 (1.015-2.983)	0.044
Unmet	44 (38.3)				
CD47					
High	54 (47.0)	3.554 (2.184-5.784)	0.000	2.869 (1.470-5.600)	0.002
Low	61 (53.0)				
TIGIT					
High	49 (42.6)	1.855 (1.166-2.953)	0.009	1.150 (0.503-2.628)	0.740
Low	66 (57.4)				
CD47/TIGIT					
High	31 (27.0)	2.987 (1.839-4.852)	0.000	1.125 (0.411-3.080)	0.819
Others	84 (73.0)				

HR, hazard ratio; CI, confidence interval; PR, parietal lobe; TM, temporal lobe.

**Figure 5:**
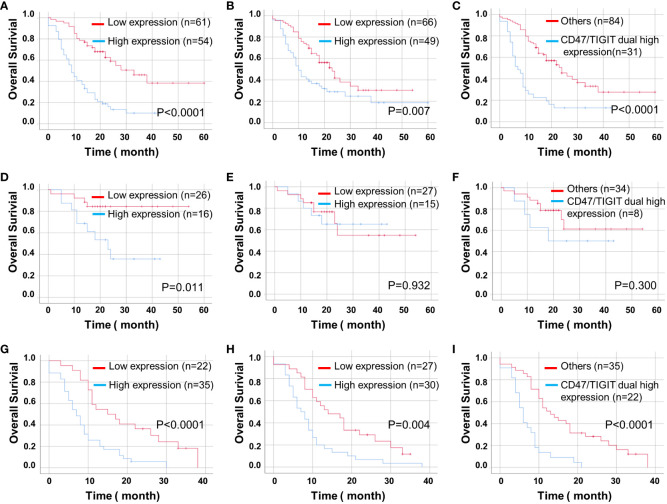
Kaplan-Meier curves of OS for CD47 and TIGIT expression and their combination in ADG (n = 115). OS for CD47 **(A)** and TIGIT **(B)** expression and their combination **(C)** in ADG. OS for CD47 **(D)** and TIGIT **(E)** expression and their combination **(F)** in astrocytoma, IDH⁃mutant (n = 42). OS for the expression of CD47 **(G)** and TIGIT **(H)** and their combination **(I)** in GBM, IDH⁃wildtype (n = 57).

We found that high expression levels of CD47 and TIGIT and double high expression of CD47/TIGIT were related to a significantly reduced OS in patients with ADG (*P* < 0.05, **Figures 5A–C**). High CD47 expression, but not TIGIT and CD47/TIGIT, significantly reduced the OS of patients with astrocytoma (*P* < 0.05, **Figures 5D–F**). In patients with GBM, high expression of CD47, TIGIT, and both CD47/TIGIT significantly decreased OS (*P* < 0.005, **Figures 5G–I**), indicating high expression levels of CD47 and TIGIT may be valuable indicators of a poor prognosis in patients with ADG.

## 4 Discussion

The new cancer classification system integrates molecular and clinical pathological diagnoses. The treatment of diffuse gliomas also requires further stratification according to the new classification. In this study, 115 cases of ADG were reclassified according to the new tumor classification, and a survival analysis of patients with ADG enrolled in the study further demonstrated that patients with GBM may experience a shorter survival than patients with astrocytoma. Patients with grade 4 astrocytoma also showed a poor prognosis. The frequencies of p*TERT* mutations were 55.7% (64/115) in ADG, 16.7% (7/42) in astroglioma, and 71.9% (41/57) in GBM. In ADG, there was no significant difference in OS between p*TERT* mutation and wild-type cases; similar results were obtained for astrocytoma and GBM. A study of the p*TERT* mutation status in GBM has indicated that while p*TERT* mutations account for about 70–80% of GBM, they are generally regarded as a late or terminal event in GBM (23), and thus their prognostic significance remains uncertain. Another study of GBM (72 patients) compared survival based on The Cancer Genome Atlas (TCGA) adult GBM cohort (24); in GBM, the p*TERT* mutant and wild-type status were not associated with OS, consistent with our results. In our study, the methylation level of *MGMT* in astrocytoma was significantly higher than that in GBM, and the *MGMT* methylation level in grade 2 was significantly higher than those of grades 3 and 4 in astrocytoma. Univariate and multivariate analyses showed that *MGMT* unmethylation may act as an independent prognostic factor for OS in ADG. A subsequent analysis of ADG subtypes revealed that patients with astrocytoma in the *MGMT* unmethylated group had a shorter OS than those in the *MGMT* methylated group. However, the difference in OS in patients with GBM between the MGMT-methylated and unmethylated groups was insignificant. In this study, the number of patients with oligodendroglioma was small, and no patients had the *MGMT* unmethylated status; therefore, survival analyses could not be performed. This result was further confirmed by another study showing that *MGMT* methylation was significantly associated with a longer PFS and OS in prospectively collected grade II gliomas treated with radiotherapy combined with temozolomide (25), suggesting that *MGMT* methylation may be a better prognostic biomarker in *IDH*-mutant LGGs.

There is an urgent need to explore new therapeutic approaches to improve the survival of patients with glioma. Although anti-PD-1/PD-L1 and Cytotoxic T-lymphocyte-associated antigen 4 (CTLA-4) monoclonal antibodies are studied extensively in glioma, most are in phase I or phase II clinical research stages (26, 27). In some clinical trials, anti-PD-L1 therapy showed slight improvements and was more effective in patients with high PD-L1 expression. However, more targets are needed due to the rapid development of drug tolerance and adverse reactions. Our study showed that CD47 and TIGIT expression levels in GBM were significantly higher than in astrocytomas and oligodendrogliomas. High expression of CD47, TIGIT, and CD47/TIGIT was all associated with poor survival in ADG and GBM, whereas high expression of CD47 was associated with shorter OS in astrocytoma.

CD47 is expressed in many tumors, such as breast cancer and anaplastic thyroid cancer, among others (28, 29). Recent studies have indicated that CD47 expression in malignant meningiomas was increased while the number of T cells was decreased, and the number of macrophages expressing CD68 was increased (30). Blocking CD47 with an anti-CD47 antibody inhibited the growth and movement of malignant meningioma cells and promoted phagocytosis mediated by macrophages (30). Liu et al. (31) showed that compared with normal controls, CD47 was more highly expressed in GBM tissues and various GBM cell lines. CD47 downregulation via siRNA suppressed invasion *in vitro*, whereas CD47 overexpression exerted the opposite effect. These results suggested CD47 may be a valuable predictor of a poor prognosis.

TIGIT is a co-inhibitory receptor that is expressed on a variety of immune cells. TIGIT interacts with different ligands, including CD155 expressed on dendritic cells, thereby inhibiting tumor killing by NK cells and antigen presentation by dendritic cells, thus weakening the anti-tumor effect of T cells (14). In recent years, TIGIT has been investigated as an important checkpoint in cancer research focused on esophageal small cell carcinoma (32) and lung adenocarcinoma (33), where TIGIT-positive patients showed a shorter OS and a lower PFS than those of TIGIT-negative patients. In the present study, TIGIT expression was high in patients with poor prognoses. High CD47/TIGIT co-expression was also associated with a poor prognosis, supporting the feasibility of using CD47 and TIGIT ICIs in treating ADG.

Our study showed an association of MGMT unmethylation with CD47 and TIGIT high expression in ADG. The addition of Temozolomide (TMZ) chemotherapy improves survival in patients with GBM containing a methylated MGMT promoter (34). A randomized phase 3 study showed that MGMT promoter methylation was also associated with better outcomes among patients with recurrent glioblastoma treated with nivolumab targeting PD-1 (35). However, for GBM patients with an unmethylated *MGMT* promoter, the benefit of TMZ is marginal and increasingly questioned. Therefore, radiotherapy is preferred when tolerable for patients with *MGMT* unmethylation (36). In contrast, for elderly, frail patients who cannot tolerate radiotherapy, the choice of anti-CD47 and TIGIT immunotherapy might be a better approach.

However, this study was affected by several limitations. Samples of the three subtypes of ADG included in the study were not balanced because the number of patients with oligodendroglioma was small, preventing evaluations of the relationship between relevant indicators and prognosis. Although we predicted the efficacy of immunotherapy using new targets for ADG, we did not include treated patients. Therefore, we were unable to draw firm conclusions regarding efficacy prediction. In addition, although we described the association of MGMT unmethylation, the expression levels of CD47 and TIGIT with patient prognosis, their therapeutic role and potential interconnections in ADG warrant to be further demonstrated by *in vivo* and *in vitro* experiments.

## 5 Conclusions

Unmethylated *MGMT* in astrocytomas and the overexpression of CD47 and TIGIT in ADG tissues are associated with a poor prognosis. Thus, patients with ADG showing high CD47 and TIGIT expression levels may benefit from anti-CD47 and TIGIT immunotherapy.

## Data availability statement

The original contributions presented in the study are included in the article/**Supplementary Materials**, further inquiries can be directed to the corresponding author/s.

## Ethics statement

The studies involving humans were approved by Ethics Committee of the First Affiliated Hospital of Shihezi University. The studies were conducted in accordance with the local legislation and institutional requirements. The human samples used in this study were acquired from a by- product of routine care or industry. Written informed consent for participation was not required from the participants or the participants’ legal guardians/next of kin in accordance with the national legislation and institutional requirements.

## Author contributions

LM: Writing – original draft, Data curation. YS: Formal analysis, Writing – review & editing. CL: Formal analysis, Writing – review & editing. BD: Writing – review & editing, Data curation. JJ: Methodology, Writing – review & editing. YC: Supervision, Writing – review & editing. HL: Resources, Supervision, Writing – original draft. LW: Investigation, Project administration, Writing – original draft.
