# Supplementary material for: MGMT unmethylation and high levels of CD47 and TIGIT indicate a poor prognosis in adult diffuse gliomas
Source: Front Immunol. 2024 Feb 9;15:1323307. doi: 10.3389/fimmu.2024.1323307 (PMC10884119; doi:10.3389/fimmu.2024.1323307)
Supplement: Supplementary file 1 [file Table_1.doc]

**Supplementary Materials**

**1 *IDH1*(R132H, R132C, and R132S) and *IDH2*R172K primers**

IDH1-F: TGGAAATCACCAAATGGCAC,

IDH1-R: TCATACCTTGCTTAATGGGTGT;

IDH2-F: GTCTTCCGGGAGCCCATCAT,

IDH2-R: AAAGCCACGAGACAGAGATGAA.

**2 Threshold for FISH tests**

| molecule change | threshold |
| --- | --- |
| 1p/19q co-deletion | 1p36/1q25 and 19q13/19p13 <0.86 |
| CDKN2A deletion | 9p21/CEP9 <0.86 |
| EGFR amplification | more than 10% of the nuclei were expressed, EGFR/CEP7 >2 |
| chromosome 7 polysomy | >20% of the nuclei showed ≥3 CEP7 signals |
| chromosome 10 monosomy | >50% of the nuclei had a CEP10 signal |

**Supplementary** **Table 1.** Clinical and molecular information of the 115 ADG according to the new edition tumors classification.

|  | **Variable** | **Astrocytoma**  **n (%)** | | |  | | **Oligodendroglioma**  **n (%)** | |  | | **Glioblastoma**  **n (%)** | |
| --- | --- | --- | --- | --- | --- | --- | --- | --- | --- | --- | --- | --- |
|  |  | **Grade 2** | **Grade 3** | **Grade 4** |  | **Grade 2** | | **Grade 3** | |  | | **Grade 4** |
| Age | ≤50 | 11 (64.7) | 10 (90.9) | 8 (57.1) |  | 7 (87.5) | | 4 (50.0) | |  | | 19 (33.3) |
|  | >50 | 6 (35.3) | 1 (9.1) | 6 (42.9) |  | 1 (12.5) | | 4 (50.0) | |  | | 38 (66.7) |
| Sex | M | 10 (58.8) | 5 (45.5) | 8 (57.1) |  | 2 (25.0) | | 1 (12.5) | |  | | 31 (54.4) |
|  | F | 7 (41.2) | 6 (54.5) | 6 (42.9) |  | 6 (75.0) | | 7 (87.5) | |  | | 26 (45.6) |
| Location | Parietal lobe | 6 (35.3) | 1 (9.1) | 7 (50.0) |  | 0 (0.0) | | 2 (25.0) | |  | | 20 (35.1) |
|  | Temporal lobe | 8 (47.1) | 6 (54.5) | 1 (7.1) |  | 5 (62.5) | | 3 (37.5) | |  | | 13 (22.8) |
|  | Frontal lobe | 0 (0.0) | 2 (18.2) | 4 (28.6) |  | 2 (25.0) | | 2 (25.0) | |  | | 9 (15.8) |
|  | Others | 3 (17.6) | 2 (18.2) | 2 (14.3) |  | 1 (12.5) | | 1 (12.5) | |  | | 15 (26.3) |
| Length (mm) | ≤50 | 12 (70.6) | 8 (72.7) | 11 (78.6) |  | 7 (87.5) | | 5 (62.5) | |  | | 38 (66.7) |
|  | >50 | 5 (29.4) | 3 (27.3) | 3 (21.4) |  | 1 (12.5) | | 3 (37.5) | |  | | 19 (33.3) |
| *TERT* promoter | Mut | 3 (17.6) | 2 (18.2) | 2 (14.3) |  | 8 (100.0) | | 8 (100.0) | |  | | 41 (71.9) |
|  | WT | 14 (82.4) | 9 (81.8) | 12 (85.7) |  | 0 (0.0) | | 0 (0.0) | |  | | 16 (28.1) |
| *MGMT* methylation | Met | 16 (94.1) | 9 (81.8) | 7 (50.0) |  | 8 (100.0) | | 8 (100.0) | |  | | 23 (40.3) |
|  | Unmet | 1 (5.9) | 2 (18.2) | 7 (50.0) |  | 0 (0.0) | | 0 (0.0) | |  | | 34 (59.7) |
| CD47 | Low | 14 (82.4) | 8 (72.7) | 4 (28.6) |  | 6 (75.0) | | 7 (87.5) | |  | | 22 (38.6) |
|  | High | 3 (17.6) | 3 (27.3) | 10 (71.4) |  | 2 (25.0） | | 1 (12.5） | |  | | 35 (61.4) |
| TIGIT | Low | 10 (58.8) | 8 (72.7) | 9 (64.3) |  | 6 (75.0) | | 6 (75.0) | |  | | 27 (47.4) |
|  | High | 7 (41.2) | 3 (27.3) | 5 (35.7 |  | 2 (25.0） | | 2 (25.0） | |  | | 30 (52.6) |

**Supplementary** **Table 2.** p*TERT* mutation and *MGMT* methylation status in ADG (n=115).

| **Group** | **Grade** | ***TERT* promoter**  **n (%)** | | ***P* value** |  | | ***MGMT* Methylation**  **n (%)** | | ***P* value** |
| --- | --- | --- | --- | --- | --- | --- | --- | --- | --- |
|  |  | **Mutation** | **Wild-type** |  |  | **Met** | | **Unmet** |  |
| Astrocytoma | 2 | 3 (42.8) | 14 (40.0) | 1.000 |  | 16 (50.0) | | 1 (10.0) | 0.014 |
|  | 3 | 2 (28.6) | 9 (25.7) |  |  | 9 (28.1) | | 2 (20.0) |  |
|  | 4 | 2 (28.6) | 12 (34.3) |  |  | 7 (21.9) | | 7 (70.0) |  |
| Oligodendroglioma | 2 | 8 (50.0) | 0 (0.0) | 0.000 |  | 8 (50.0) | | 0 (0.0) | 0.000 |
|  | 3 | 8 (50.0) | 0 (0.0) |  |  | 8 (5 0.0) | | 0 (0.0) |  |
| Astrocytoma |  | 7 (10.9) | 35 (68.6) | 0.000 |  | 32 (45.1) | | 10 (22.7) | 0.000 |
| Oligodendroglioma |  | 16 (25.0) | 0 (0.0) |  |  | 16 (22.5) | | 0 (0.0) |  |
| Glioblastoma | 4 | 41 (64.1) | 16 (31.4) |  |  | 23 (32.4) | | 34 (77.3) |  |

**Supplementary** **Table 3.** Expression of CD47 and TIGIT in ADG tissues (n=115).

| **Group** | **Grade** | **CD47 expression**  **n (%)** | | ***P* value** |  | | **TIGIT expression**  **n (%)** | | ***P* value** |
| --- | --- | --- | --- | --- | --- | --- | --- | --- | --- |
|  |  | **Low** | **High** |  |  | **Low** | | **High** |  |
| Astrocytoma | 2 | 14 (53.8) | 3 (18.75) | 0.006 |  | 10 (37.0) | | 7 (46.7) | 0.755 |
|  | 3 | 8 (30.8) | 3 (18.75) |  |  | 8 (29.7) | | 3 (20.0) |  |
|  | 4 | 4 (15.4) | 10 (62.5) |  |  | 9 (33.3) | | 5 (33.3) |  |
| Oligodendroglioma | 2 | 6 (46.2) | 2 (66.7) | 0.522 |  | 6 (50.0) | | 2 (50.0) | 1.000 |
|  | 3 | 7 (53.8) | 1 (33.3) |  |  | 6 (50.0) | | 2 (50.0) |  |
| Astrocytoma |  | 26 (42.6) | 16 (29.6) | 0.004 |  | 27 (40.9) | | 15 (30.6) | 0.075 |
| Oligodendroglioma |  | 13 (21.3) | 3 (5.6) |  |  | 12 (18.2) | | 4 (8.2) |  |
| Glioblastoma | 4 | 22 (36.1) | 35 (64.8) |  |  | 27 (40.9) | | 30 (61.2) |  |
